# Molecular Modeling Unveils the Effective Interaction of B-RAF Inhibitors with Rare B-RAF Insertion Variants

**DOI:** 10.3390/ijms241512285

**Published:** 2023-07-31

**Authors:** Maria Chiara Scaini, Luisa Piccin, Davide Bassani, Antonio Scapinello, Stefania Pellegrini, Cristina Poggiana, Cristina Catoni, Debora Tonello, Jacopo Pigozzo, Luigi Dall’Olmo, Antonio Rosato, Stefano Moro, Vanna Chiarion-Sileni, Chiara Menin

**Affiliations:** 1 Immunology and Molecular Oncology Unit, Veneto Institute of Oncology IOV-IRCCS, 35128 Padua, Italy; mariachiara.scaini@iov.veneto.it (M.C.S.); stefania.pellegrini@iov.veneto.it (S.P.); cristina.poggiana@iov.veneto.it (C.P.); cristina.catoni@iov.veneto.it (C.C.); debora.tonello@iov.veneto.it (D.T.); antonio.rosato@unipd.it (A.R.); chiara.menin@iov.veneto.it (C.M.); 2 Melanoma Unit, Oncology 2 Unit, Veneto Institute of Oncology IOV-IRCCS, 35128 Padua, Italy; luisa.piccin@iov.veneto.it (L.P.); jacopo.pigozzo@iov.veneto.it (J.P.); vanna.chiarion@iov.veneto.it (V.C.-S.); 3 Molecular Modeling Section (MMS), Department of Pharmaceutical and Pharmacological Sciences, University of Padova, 35131 Padua, Italy; davide.bassani.1@studenti.unipd.it; 4 Anatomy and Pathological Histology Unit, Veneto Institute of Oncology IOV-IRCCS, 35128 Padua, Italy; antonio.scapinello@iov.veneto.it; 5 Soft-Tissue, Peritoneum and Melanoma Surgical Oncology Unit, Veneto Institute of Oncology IOV-IRCCS, 35128 Padua, Italy; 6 Department of Surgery, Oncology and Gastroenterology (DISCOG), University of Padua, 35128 Padua, Italy

**Keywords:** ligand-based homology modeling, molecular docking calculation, BRAF rare mutations, advanced melanoma, targeted therapy, liquid biopsy

## Abstract

The Food and Drug Administration (FDA) has approved MAPK inhibitors as a treatment for melanoma patients carrying a mutation in codon V600 of the BRAF gene exclusively. However, BRAF mutations outside the V600 codon may occur in a small percentage of melanomas. Although these rare variants may cause B-RAF activation, their predictive response to B-RAF inhibitor treatments is still poorly understood. We exploited an integrated approach for mutation detection, tumor evolution tracking, and assessment of response to treatment in a metastatic melanoma patient carrying the rare p.T599dup B-RAF mutation. He was addressed to Dabrafenib/Trametinib targeted therapy, showing an initial dramatic response. In parallel, in-silico ligand-based homology modeling was set up and performed on this and an additional B-RAF rare variant (p.A598_T599insV) to unveil and justify the success of the B-RAF inhibitory activity of Dabrafenib, showing that it could adeptly bind both these variants in a similar manner to how it binds and inhibits the V600E mutant. These findings open up the possibility of broadening the spectrum of BRAF inhibitor-sensitive mutations beyond mutations at codon V600, suggesting that B-RAF V600 WT melanomas should undergo more specific investigations before ruling out the possibility of targeted therapy.

## 1. Introduction

Approximately 50% of cutaneous melanomas harbor an activating mutation in the BRAF gene [1], making it an ideal target for therapy in advanced stages. Over 90% of the observed B-RAF mutations typically occur in codon 600, located within the kinase domain of the protein. These mutations result in the replacement of valine, primarily with glutamic acid, and more rarely with lysine, aspartic acid, or arginine (p.V600E/K/D/R).

These V600 mutations induce a heightened intrinsic activity of B-RAF as a monomer and are referred to as class I BRAF mutations.

Advanced melanoma patients carrying a V600 B-RAF mutation are the only cases for which the Food and Drug Administration (FDA) has approved MAPK inhibitors as treatment (https://www.fda.gov/drugs, last accessed on 27 July 2023). However, approximately 3–5% of melanomas harbor rare mutations in the BRAF gene outside the V600 codon (non-V600 mutations). Some of these rare mutations, referred to as “class II”, act as RAS-independent active dimers inducing intermediate kinase activity, while others, referred to as “class III”, disclose a RAS-dependent impaired, and very low, kinase activity. Several preclinical studies and case reports [2,3,4,5] have evaluated the potential benefit of FDA-approved MAPK inhibitors in class II and III BRAF-mutated melanoma, often with conflicting results. 

In the latest decades, the drug discovery pipelines in both academic and industrial environments have proven to be heavily characterized by the implementation of computational approaches [6,7,8]. Indeed, such techniques have been demonstrated to give a boost to the molecular candidates’ development process, drastically reducing the time and money that would have been required to experimentally assay all the properties that have been evaluated in the virtual environment handled by expert computational scientists [9]. Techniques such as Molecular Docking [10], Molecular Dynamics (MD) [11], quantum mechanical (QM)-related calculations [12], and machine learning (ML) [13], which all fall into the domain of the so-called Computer-Aided Drug Design (CADD) science [14], have been regularly and successfully applied in the drug discovery world to prioritize better candidates for a desired biological outcome [15].

Considering the huge interest that the protein kinase B-RAF represents from a therapeutic point of view, it is clear that computational approaches have also been applied in the discovery process for the inhibition of this target, and they proved to be proficient in their tasks. One very noticeable example is Vemurafenib, a small molecule approved for the treatment of late-stage melanomas, specifically active against the B-RAF V600E mutant [16,17]. For the discovery of this molecule, computer-based methods have been used and combined with experimental techniques in the so-called “pipeline of fragment-based drug discovery” (FBDD). Computational approaches have also been used for the discovery of other B-RAF inhibitors, such as Dabrafenib [18] and Encorafenib [19].

Molecular docking is one of the most applied methods in drug discovery nowadays [10] and it consists of creating the most reasonable conformations of a chemical entity bound to another in order to form a stable complex [20]. Specifically, it is primarily used to predict the best conformations (named “poses”) of ligand when binding to a biological target (protein or nucleic acid). The molecular docking technique is based on the iterative operation of two main actors: the search algorithm and the scoring function. The first has the role of creating a series of conformations of the ligand bound to its target, while the second has the role of evaluating each of these conformations for geometrical and energetical parameters, prioritizing the poses that are more likely to form a stable complex with the target [21]. At the end of the run, a defined number of the most proper conformational solutions are given to the user. A great variety of programs are nowadays available for molecular docking, exploiting different search algorithm-scoring function combinations. Among these are the programs GOLD [22] (a genetic algorithm developed by CCDC), Glide [23] (a systematic docking program developed and distributed by Schrödinger), Autodock [24] (a free-to-use program created by the Scripps Research Institute) and PLANTS [25] (an Ant-Colony-Optimization program developed by the University of Tübingen). Molecular docking has historically been successfully implemented in the computational drug discovery pipelines, as abundantly assessed in the literature [26,27,28,29].

Moving a little bit outside of the approved drug space, CADD techniques are routinely exploited by academic and industrial groups all over the world to find novel, potent, and safe regulators of B-RAF. Luo et al. exploited a molecular docking-based virtual screening that led to the identification of micromolar small molecule inhibitors of this target [30]. Molecular docking was also implemented by Dong et al. to rationalize the structure-activity relationship (SAR) among a series of 5-phenyl-1H-pyrazol derivatives, which are active against the B-RAF V600E mutant in the high-nanomolar range [31]. Last but not least among the presented examples, always configured from a rationalization perspective, Marini et al. exploited computational tools for the investigation of the behavior discrepancy between several proteolysis-targeting chimeras (PROTACs) of interest [32].

Here, we report a molecular modeling analysis of two rare class II BRAF mutations characterized by the insertion of a threonine (c.1795_1797dup p.T599dup) or valine (c.1794_1795insGTT p.A598_T599insV) into the activation segment of the B-RAF kinase domain. Starting from the observation of a clinical response to targeted therapy in a patient with B-RAF T599dup mutant melanoma, we applied ligand-based homology modeling to both mutant proteins using a B-RAF-V600E X-ray structure as a template. Indeed, computer-based approaches have been widely used in drug discovery and development [33,34,35,36], both to speed up the identification of promising therapeutically relevant molecules [37,38,39] and to rationalize experimental outcomes [40,41,42]. This approach allowed for a three-dimensional structure of both the rare B-RAF mutated proteins with a pocket accessible to Dabrafenib and V600E. Moreover, a molecular docking calculation was used to validate the effectiveness of Dabrafenib’s interaction with the p.T599dup and p.A598_T599insV B-RAF proteins, demonstrating that both mutations can be efficiently recognized by this drug in a very similar manner to class I BRAF variants. We also provided additional evidence of the effective inhibition of the p.T599dup B-RAF-mutant protein by following the trend of the BRAF-mutant clone in patient blood through a longitudinal liquid biopsy-based approach, corroborating data coming from CT scans and immunohistochemistry (IHC).

## 2. Results

### 2.1. Disease Evolution, Clinical Assessment, and Integrated Analyses

In October 2019, a 57-year-old patient, in follow-up for a pT3aN0M0 nodular melanoma diagnosed in July 2012, presented new asymptomatic pleural nodes on a computed tomography (CT) scan (Supplementary Figure S1). BRAF analysis of the primary melanoma by real-time PCR (rtPCR) using the EasyPGX Ready BRAF system (Diatech Pharmacogenetics), specific only for BRAF V600 variants, did not detect any mutations.

In November 2019, the patient started anti-PD-1 therapy, which was discontinued in September 2020 due to pleural, nodal, and liver progressive disease (PD, according to iRECIST criteria) and followed, from October 2020, by anti-CTLA4 monoclonal antibody administration (Supplementary Figure S1). After 2 months, this treatment was combined with an anti-PD1 agent because of a further unequivocal PD. Until that time Patient’s Eastern Cooperative Oncology Group Performance Status (ECOG PS) was 0 and lactate dehydrogenase (LDH) levels were below the upper limit of normal (ULN).

**Figure 1:**
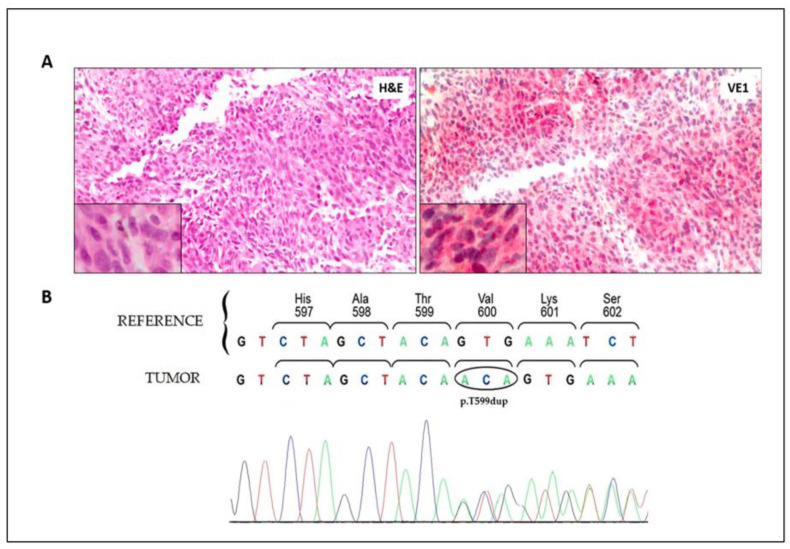
**B-RAF immunohistochemistry and sequencing of the metastatic pleural lesion**. (**A**) Staining with hematoxylin and eosin (H&E) and anti-B-RAF V600E monoclonal antibody (VE1) shows the diffuse presence of melanoma cells with cytoplasmic and nuclear localization of the B-RAF mutant protein. Original magnification: 20× (enlargement: 40×). (**B**) Sequencing electropherogram of the BRAF exon 15 showing the frameshift due to the duplication of codon 599 (c.1795_1797dup) causing the insertion of a threonine amino acid in the B-RAF protein (p.T599dup).

In March 2021, the patient’s clinical conditions worsened (ECOG PS, 2) due to a symptomatic and rapid clinical progression (increasing dyspnea) with pleural effusion appearance, liver and nodal metastases growth, and evidence of suspected pancreatic lesions. S100 protein circulating levels were 2.57 µg/L (reference values 0–0.15 µg/L), while LDH was still in range. At this time (Supplementary Figure S1), a new tumor biopsy coming from a pleural lesion was analyzed for the BRAF V600 mutation both by rtPCR (confirming the negative results obtained from the primary tumor) and by IHC (VE1 antibody specific for the V600E-B-RAF protein) that showed an unexpected positive staining in almost all melanoma cells (Figure 1A). BRAF exon 15 was then sequenced, and a rare BRAF variant, c.1795_1797dup (p.T599dup), was identified (Figure 1B).

**Figure 2:**
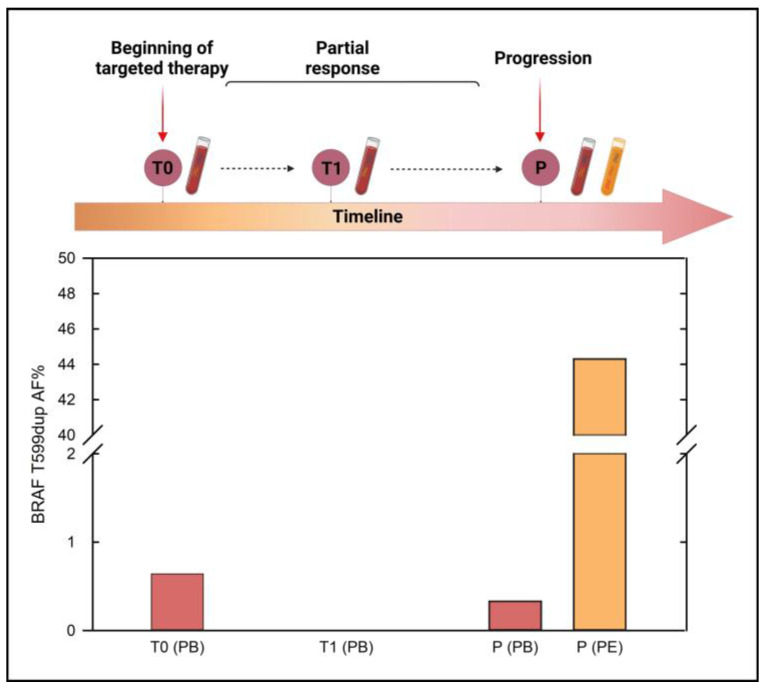
**BRAF MAF (%) detected in liquid biopsy samples collected at different time points**. T0: before starting the targeted therapy (April 2021); T1: month 6 follow-up during the clinically disease-free period (October 2021); P: progression (February 2022). Abbreviations: PB, peripheral blood; PE, pleural effusion. The timeline was created with BioRender (https://biorender.com/); the plot was performed using Sigma Plot version 14.0 (Systat Software, San Jose, CA, USA).

Noteworthy, the mutation was confirmed by droplet digital PCR (ddPCR) and NGS on both the primary tumor and the metastasis, following a later re-examination performed after the last PD. More in detail, the customized ddPCR assay identified the presence of BRAF p.T599dup at a mutant allele fraction (MAF) of 34% and 48% in the primary and metastatic tissues, respectively (Supplementary Figure S2). These findings were independently validated by the NGS panel, which detected MAFs of 28.6% and 44.2% for the primary and metastatic tissues, respectively. Following these results, a subsequent IHC review of the primary tumor identified a mild focal positivity consistent with the presence of the B-RAF mutation (Supplementary Figure S3). As B-RAF p.T599dup was reported to be sensitive to Dabrafenib [43,44], the combined treatment with Dabrafenib/Trametinib was started in April 2021. Five days after starting the new treatment, the patient’s ECOG PS turned to 0, becoming able to perform activities of daily living (ADL) and instrumental activities of daily living (IADL) independently again. The first CT scan tumor assessment (August 2021) confirmed a partial response (PR, RECIST 1.1 criteria) with a decrease above all of pleural lesions, effusions, and liver metastases. Moreover, the S100 value decreased from 2.57 µg/L to 0.37 µg/L. Accordingly, during the response to targeted therapy, the BRAF-mutant allele fraction, detectable in the cell-free circulating tumor DNA (ctDNA) at the start of treatment (MAF of 0.64%), became undetectable. In February 2022, the patient’s clinical condition worsened again, as evidenced by a CT scan of pleural, liver, and brain progression. In parallel, the ctDNA analysis, performed on both plasma and pleural effusion, detected a rebound of the BRAF MAF (Figure 2). More in detail, the longitudinal tracking of ctDNA, performed by NGS just before the start of targeted therapy (baseline, T0), six months later (T1), and at progression (P), showed that BRAF p.T599dup became undetectable during response and rebounded at progression both in plasma (MAF 0.33%) and in pleural effusion ctDNA (MAF 44.3%). Moreover, the NGS analysis identified several different single nucleotide variants (SNVs), four of which are annotated in the Catalogue of Somatic Mutations in Cancer (COSMIC) (Supplementary Figures S1 and S4). Interestingly, the BRAF p.T599dup rebound overlapped with that of the PPP6C p.H151Y, putatively responsible for the resistance to targeted therapy [45,46,47,48]. Indeed, at progression, PPP6C p.H151Y, not detected at T0 and T1, showed an allele frequency of 0.8% and 72.6% in plasma and pleural effusion, respectively. The targeted therapy was then discontinued, and after 4 cycles of temozolomide (best response: PD according to RECIST 1.1), in August 2022, the patient shifted to immunotherapy treatment again, and radiation therapy on the lung was performed. The last CT scan performed (May 2023) showed unequivocal PD on the liver, pleura, nodes, and soft tissues, and evaluation for a possible new chemotherapy line is currently ongoing.

**Figure 3:**
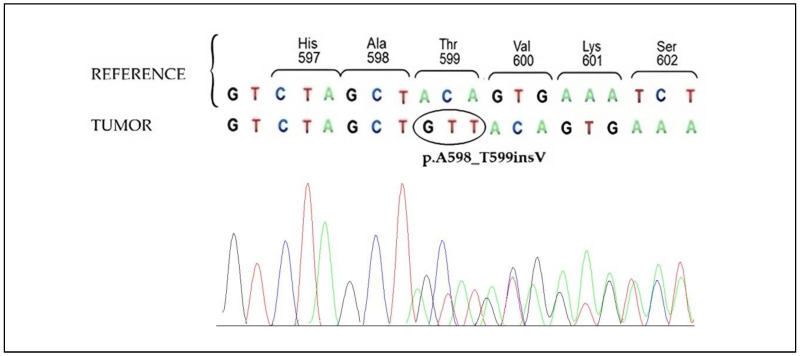
**BRAF sequencing analysis of pT2a primitive melanoma**. Electropherogram of the BRAF exon 15 showing the frameshift due to the nucleotide triplet insertion before the codon 599 (c.1794_1795insGTT) causing the insertion of a valine amino acid in the B-RAF protein (p.A598_T599insV).

In the meantime, another B-RAF insertion variant (p.A598_T599insV) (Figure 3) was identified in a patient with a pT2aN0M0 melanoma, raising the question of whether it might also be responsive to Dabrafenib. Nevertheless, this second patient never developed disease progression and is still receiving only regular follow-up.

### 2.2. Molecular Modeling

**Figure 4:**
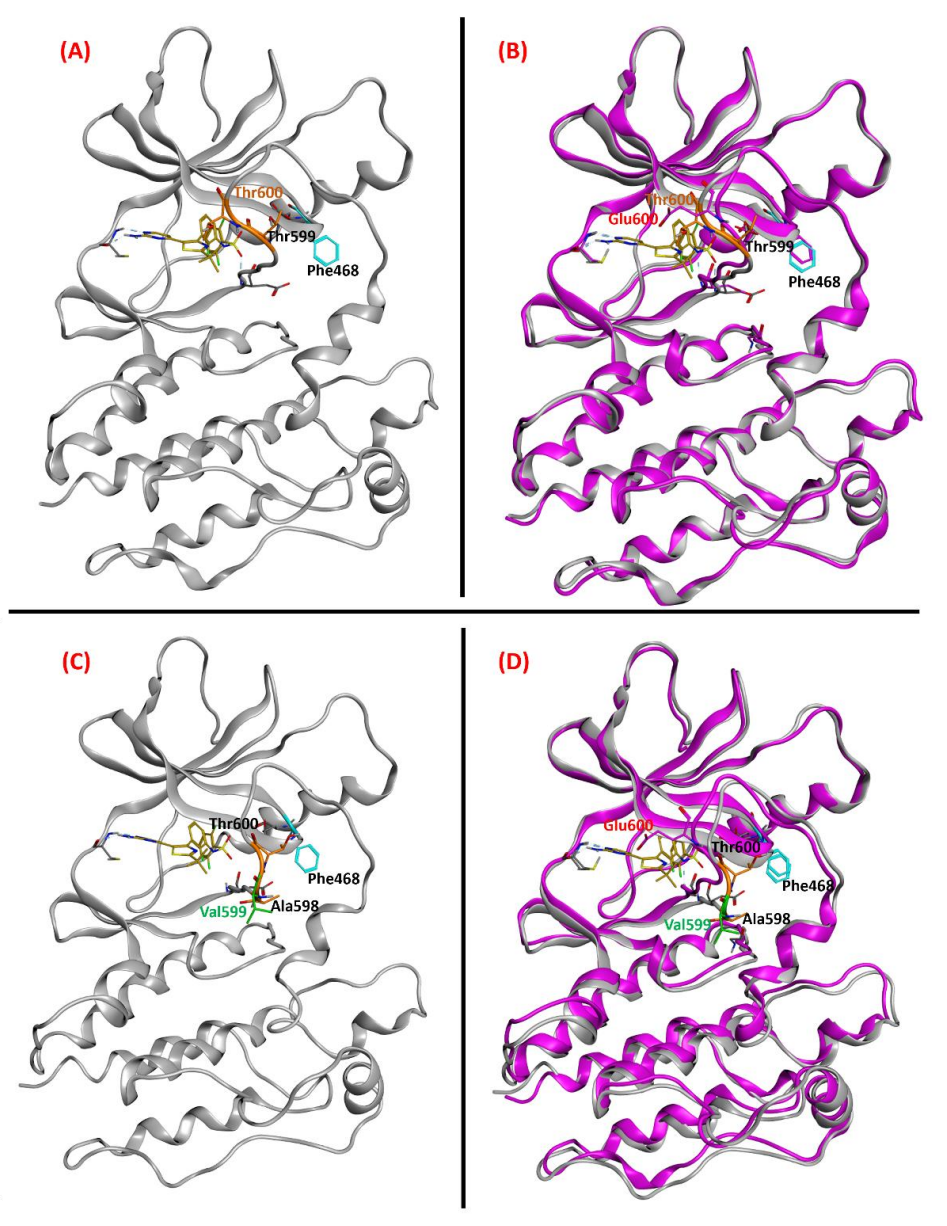
(Panel (**A**)) Ligand-based homology model for the T599dup B-RAF variant complexed with Dabrafenib (from PDB: 4XV2, colored in gold). The T599 and the newly inserted T600 residues are colored orange and labeled in black and orange, respectively. (Panel (**B**)) Superposition of the backbones of the T599dup B-RAF/Dabrafenib ligand-based homology model with the reference crystal structure of B-RAF V600E (PDB code: 6P7G). The E600 residue of the V600E variant is colored magenta and labeled in red. The RMSD of the backbones of the two superposed structures was 0.60 Å. (Panel (**C**)) Ligand-based homology model for the A598T599_insV B-RAF variant complexed with Dabrafenib. The A598 and the T600 residues are colored orange and labeled in black; the newly inserted V599 residue is colored and labeled in green. (Panel (**D**)) Superposition of the backbones of the A598_T599_insV B-RAF/Dabrafenib ligand-based homology model with the reference crystal structure of B-RAF V600E. The RMSD of the backbones of the two superposed structures was 0.96 Å. The hydrogen bonds are highlighted with cyan-colored sticks, whose thickness is proportional to their strength. All the images represented in the panels were created and rendered with MOE.

Our goal was to prove that Dabrafenib could successfully interact with and inhibit the p.T599dup and the p.A598_T599insV B-RAF mutations in a way similar to the one in which it inhibits the well-known p.V600E mutant B-RAF. Both the p.T599dup and the p.A598_T599insV B-RAF mutations lack an experimentally resolved structure, so a ligand-based homology modeling [49] approach was adopted in order to create reasonable models for them. The MOE homology modeler tool was implemented for this task, and the methodology comprised conformational optimization and refinement, with side chains modeled with data coming from the well-sampled database generated through the LowModeMD technique, which couples short isothermal MD simulations with subsequent all-atom energy minimizations [50]. The sequence used to build the B-RAF mutant proteins was manually created starting from the WT sequence available on UniProt [51], access code P15056. Based on our and previous observations of sensitivity to B-RAF inhibitors of p.T599dup B-RAF mutant melanomas [43,52] and the principle that two proteins with a high sequence similarity also have a very similar three-dimensional conformation [53], we applied a homology modeling approach using as a template the Protein Data Bank X-ray structure with code 6P7G [54] because it had the highest resolution among the B-RAF V600E crystals due to the entire activation loop being experimentally resolved (Supplementary Figure S5). The advantage of ligand-based homology modeling lies in the fact that the obtained models have their amino acid side chains already oriented to allow the ligand to be allocated in the interaction site, thus supporting experimental evidence. We created a completely resolved structure of B-RAF V600E with Dabrafenib placed in the binding site (from the X-ray complex with PDB code 4XV2, in which B-RAF V600E is complexed with Dabrafenib but the activation loop is not resolved). The final complexes were compared to the initial for backbone superposition (Figure 4A,B). Then, we used the same approach to create a p.A598_T599insV B-RAF three-dimensional model with an active loop suited to complex with Dabrafenib (Figure 4C,D). The RMSD of the backbones of the T599dup and the A598_T599insV B-RAF/Dabrafenib superposed structures was 0.60 Å and 0.96 Å, respectively.

To verify the stability of Dabrafenib bound to the active pocket of the ligand-based homology model created for the p.T599dup and p.A598_T599insV B-RAF proteins, we used the molecular docking technique.

**Figure 5:**
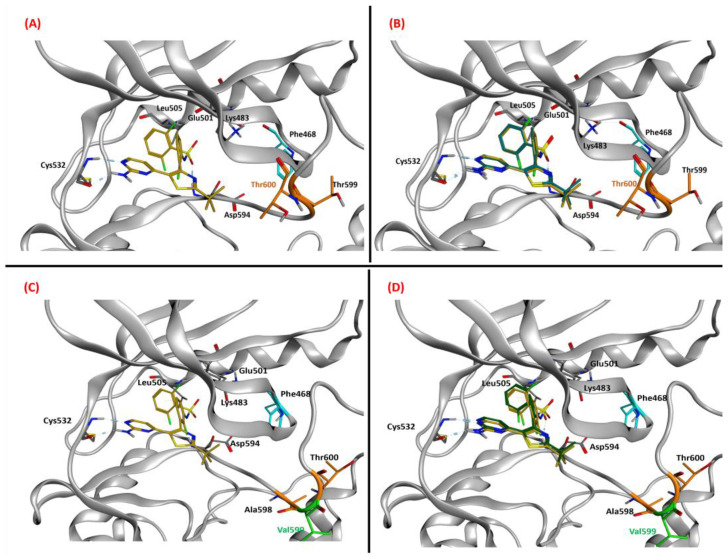
(Panel (**A**)) Dabrafenib (colored in gold) in the binding pocket of the T599dup ligand-based homology model. The T599 and the newly inserted T600 residues are colored in orange and labeled in black and orange, respectively. (Panel (**B**)) Superposition of the T599dup B-RAF/Dabrafenib ligand-based homology model with the best docking pose obtained with Glide (colored in dark green). The RMSD between the two Dabrafenib poses was 0.22 Å. (Panel (**C**)) Dabrafenib in the binding pocket of the A598_T599_insV ligand-based homology model. The A598 and the T600 residues are colored orange and labeled in black; the newly inserted V599 residue is colored and labeled in green. (Panel (**D**)) Superposition of the A598_T599_insV B-RAF/Dabrafenib ligand-based homology model with the best docking pose obtained with Glide. The RMSD between the two Dabrafenib poses was 0.38 Å. The hydrogen bonds are highlighted with cyan-colored sticks, whose thickness is proportional to their strength. All the images represented in the panels were created and rendered with MOE.

In this study, we implemented Glide as the software for molecular docking, using Glide-SP as the scoring function. In each docking experiment, 25 poses of Dabrafenib complexed within the B-RAF and B-RAF mutants were obtained. These conformations were then filtered to eliminate those eventually presenting clashes or unfavorable electrostatic interactions with the protein. Then, each of the poses was compared with the crystallographic conformation of Dabrafenib (coming from the PDB 4XV2 crystal), and the RMSD of their coordinates was computed. The pose with the highest overlap with the crystallographic coordinates of Dabrafenib has been prioritized, and in this case, the pose corresponded to the conformation that was top-ranked by GlideScore (Figure 5A–D). This relation between superimposability and docking score is not granted; considering that relying on docking scores has been demonstrated to be misleading by abundant scientific literature [55,56,57], we chose to base our pose prioritization approach just on the closeness to the experimental crystallographic coordinates of Dabrafenib. Indeed, for the p.T599dup and the p.A598_T599insV B-RAF variants, the RMSD of the best pose produced by Glide was 0.22 Å and 0.38 Å, respectively. In conclusion, the computational protocol successfully provided a molecular panorama of the effectiveness of Dabrafenib binding on the B-RAF mutations analyzed in our study, demonstrating that both of these entities can be efficiently recognized and inhibited by Dabrafenib in a very similar manner in which it inhibits the V600E mutant one.

## 3. Discussion

This work allowed us to shed light through an in silico approach on the conformational changes that may justify the efficacy of a B-RAF inhibitor, such as Dabrafenib, designed to bind to the B-RAF V600-mutant form, even in the presence of other nearby V600 mutations, such as p.T599dup or p.A598_T599_insV. Moreover, our data can be viewed as an advancement in relation to other studies that have already reported the efficacy of Dabrafenib for patients with tumors carrying this type of mutation [43,44,52,58,59]. What sets our work apart is that the clinical response is corroborated in silico and monitored by molecular analyses. Indeed, for the patient carrying the p.T599dup mutation, a liquid biopsy approach was available, demonstrating that longitudinal monitoring of the BRAF mutant allele fraction by liquid biopsy is a useful tool to identify tumor progression and track disease evolution. It is noteworthy that both the response and progression could have been inferred from the ctDNA trend and that the liquid biopsy data corroborated the hypothesis of a correct binding between Dabrafenib and the mutant B-RAF, which was also suggested by the results coming from IHC and downstream molecular modeling. In the era of personalized medicine, where targeted drugs have gained paramount importance, these findings prompt us to consider the potential clinical impact of treating tumors that harbor different SNVs near the specific hotspot of a targeted drug with the same compound. Tumors with these SNVs may potentially exhibit similar clinical effects to those harboring the specific mutation targeted by the drug. Noteworthy, this study emphasizes the potential advantages of an integrated approach, gathering sequencing, and liquid biopsy longitudinal tracking. Liquid biopsy has emerged as a promising companion diagnostic tool for targeting tumor heterogeneity with prognostic and/or predictive potential using specific cut-offs [60,61]. Although this study presents a single case, which is not sufficient to establish a routine workflow, it demonstrates how a simple and non-invasive approach enables close monitoring of disease progression. Real-time monitoring could aid in determining the optimal timing for transitioning to a different line of therapy or discontinuing the current treatment.

The role of in silico tools has become evident not only in drug design, where their contribution is unquestionable, but also in supporting the selection of specific drugs and determining the appropriate timing for their administration [33,62]. These findings also open up the possibility of broadening the spectrum of B-RAF inhibitor-sensitive variants beyond mutations at codon V600. Based on the data we collected and those already reported in the literature, B-RAF V600 WT melanomas should undergo more specific investigations before ruling out the possibility of being treated with targeted therapy. The computational analysis here presented supports the experimental data collected on B-RAF, and it is also an introduction to a much broader computational study on the effect of mutations on kinase-ligand recognition, with a strong emphasis on the B-RAF mutants reported in this manuscript. This work involves not just molecular docking and model refinement but also extensive use of MD simulations as the primary post-docking strategy. Moreover, the novel approach known as Thermal Titration Molecular Dynamics (TTMD), which was recently developed and validated by our group [39,63], will be exploited for pose prioritization. The results of this ongoing, extensive study will be useful in better understanding the interaction mechanics beneath mutant-ligand recognition.

## 4. Conclusions

This work highlights the importance of identifying non-V600, rare B-RAF variants that may still be responsive to treatment with B-RAF inhibitors. Moreover, this work emphasizes the usefulness of defining a combined in silico/molecular approach capable of identifying a suitable therapy and following the response over time. Indeed, monitoring treatment response is essential to determining the benefit of new therapies and avoiding the prolonged use of ineffective and potentially toxic treatments. It was valuable to identify a cross-reaction of the VE1 monoclonal antibody, specific for the p.V600E mutation of B-RAF, with the mutations under investigation. Although it is evident that the affinity is lower, these findings are important. While it may not be completely in agreement with other published data [64], our work paves the way for more in-depth studies that can define a more rational use and accurate interpretation of the immunohistochemistry data. Finally, our combined approach showed the potential to expand the cohort of patients putatively treatable with B-RAF inhibitors, which could be of great importance to improving the management of advanced melanoma.

## 5. Materials and Methods

### 5.1. Sample Collection, Tissue and cfDNA Analyses

Qualitative real-time PCR (rtPCR) was performed to analyze the BRAF gene in both the primary melanoma tissue and the pleural lesion biopsy. The EasyPGX Ready BRAF kit (Diatech Pharmacogenetics, Jesi AN, Italy) was used for this analysis, following the manufacturer’s instructions. Then, the BRAF exon 15 status was further assessed by Sanger sequencing. Briefly, DNA was extracted from the enriched tumor area selected by the pathologist from the formalin-fixed paraffin-embedded (FFPE) tissue. BRAF exon 15 was amplified using forward 5′-TCATAATGCTTGCTCTGATAGGA-3′ and reverse 5′-GGCCAAAATTTTAATCAGTGGA-3′ primers and sequenced using the Big Dye Terminator v1.1 Cycle Sequencing Kit (Applied Biosystems, Austin, TX, USA) on a 96-capillary sequencer (AB3730xl Genetic Analyzer).

B-RAF immunohistochemistry (IHC) staining was performed on a Ventana Benchmark ULTRA platform (Roche, Monza, Italy) using Ventana^®^ antibody mutation-specific monoclonal antibody (VE1) in combination with the Ventana ultraView universal alkaline phosphatase red detection kit according to the manufacturer’s instructions. Briefly, 4 µM thick sections were deparaffinized and treated for antigen retrieval. After incubation with mouse monoclonal antibodies, tissue sections were treated with peroxidase inhibitors and buffers containing a cocktail of HQ-labeled antibodies and HRP-conjugated anti-HQ antibodies. Then, slides were counterstained with Hematoxylin II. Each stain had internal positive and negative controls. B-RAF IHC evaluation was considered to indicate a positive phenotype when cytoplasmic background staining was present [65].

Serial blood sampling was set at different time points to longitudinally track disease evolution and response to targeted therapy through liquid biopsy. Peripheral blood was collected before starting the therapy (T0), after 6 months (T1), and at the time of progression (P). Pleural effusion was collected from the thoracentesis performed 2 weeks after progression. Blood samples were collected in Streck Cell-Free DNA BCT tubes (Streck, La Vista, NE, USA) for circulating cell-free DNA (cfDNA) analysis by next-generation sequencing (NGS) and droplet digital PCR (ddPCR, Bio-Rad Laboratories, Hercules, CA, USA). Plasma and pleural effusion were centrifuged twice prior to being stored at −80 °C. cfDNA was isolated from stored plasma/pleural effusion by the QIAamp Circulating Nucleic Acid Kit (Qiagen, Hilden, Germany) and quantified on a Qubit Fluorometric Quantitation System 1.0 (Thermo Fisher Scientific, Waltham, MA, USA), following the manufacturer’s instructions. A quality control (QC) test was also performed with the 4200 TapeStation System (cell-free DNA ScreenTape Assay, Agilent Technologies, Santa Clara, CA, USA).

Droplet digital PCR reactions were performed in duplicate in a 20 µL reaction mix containing 1× droplet PCR supermix, 250 nM of each probe, 450 nM primers, and 7 µL of FFPE DNA (derived from primary and metastatic tumor tissue samples). Samples were analyzed with a custom assay for tracking the BRAF p.T599dup mutation (UniqueAssayID: dHsaMDS440680521; BioRad, Hercules, CA, USA). Droplets were generated and analyzed using the QX200 system (BioRad, Hercules, CA, USA). Thermal conditions were as follows: 1 cycle of 95 °C for 10 min, 40 cycles of 94 °C for 30 s and 55 °C for 1 min followed by 98 °C for 10 min and a 4 °C infinitive hold. Positive-, negative-, and no-template controls were included in each run. The data were acquired and analyzed by QuantaSoft analysis software version 1.7.4 (BioRad, Hercules, CA, USA). The MAF was expressed in percentage and defined as the number of copies of mutant DNA/total number of copies of mutant DNA plus wild-type DNA.

The SureSelect All-In-One NGS custom panel (Agilent Technologies; see [66] for panel specifics) was performed on both the primary/metastatic tumor tissue samples and plasma-/pleural effusion- derived ctDNA. Alignment and variant calling were assessed through the SureCall software v.4.2 (Agilent Technologies), with interpretation and prioritization by Alissa Interpret Analysis Software v.5.3.4 (Agilent Technologies).

### 5.2. Molecular Modeling

The basic molecular modeling operations, as well as the preparation of both proteins and ligands for computational manipulation, were carried out with the Molecular Operating Environment suite (MOE, version 2019.01 [67]). The hardware used consisted of 12 CPUs (with Intel Xeon E5-1620 Linux workstation.

Before any computational manipulation of biological and chemical objects, a proper preparation procedure is mandatory for molecular modeling [68]. In our case, the Protein Data Bank was searched (latest access 8 March 2022), and the structure with code 6P7G [69] (method: X-ray diffraction, resolution: 2.65 Å) was selected. This was then imported into the main MOE window and subjected to a preparation protocol consisting of the implementation of different tools of the MOE suite. First, only chain B, which is totally experimentally resolved, was retained for the subsequent steps. Second, the “structure preparation” tool was exploited to select the orientation of the amino acid side chains based on their crystallographically derived “occupancy”. Then, the “Protonate 3D” application was used to assign to each amino acid the proper protonation state (setting the pH parameter to 7.4), and, finally, the added hydrogens were minimized under the AMBER10:EHT [70] force field implemented in MOE. The Dabrafenib molecule was prepared for molecular docking by exploiting the Wash tool implemented in MOE. Only the most abundant protomer at pH 7.4 was considered, and its 3D coordinates were rebuilt by cyclic 3D embedding based on distance geometry, followed by refinement.

The computational technique of ligand-based homology modeling was adopted, as no experimental and sufficiently reliable structure was available for the complex between Dabrafenib and the B-RAF mutants of interest [71]. As already mentioned, this approach is based on the principle that, if two proteins are highly similar in sequence, they will also be very superimposable in their three-dimensional conformation [53] The reference to build the models on was the prepared structure with PDB code 6P7G. The conformation of Dabrafenib inside the pocket of the protein models was taken from the PDB 4XV2 crystal [72] (method: X-ray diffraction, resolution: 2.50 Å, which underwent the same preparation procedure as 6P7G), after having superposed its backbone to the 6P7G one. With this method, a totally resolved complex of B-RAF V600E was created, taking the backbone from the X-ray structure with PDB 6P7G and Dabrafenib, placed in the binding site, from the PDB complex with code 4XV2. This system was now suitable for the ligand-based homology modeling approach. The sequences used to generate both the T599dup and the p.A598_T599insV B-RAF mutants were manually built starting from the WT one (available on UniProt [51] access code P15056).

The homology modeling was executed with the dedicated homology modeler tool implemented in MOE. Specifically, ligand-based homology modeling starts by fixing the coordinates of the ligand around which the model will be created. Then, the geometry of the model amino acids is modeled according to their similarity to the ones owing to the template, and atoms whose coordinates are copied from template atoms carry along with them all attributes that affect the following energy minimization steps. In cases in which loops or deletions are present, they are retreated from high-resolution chains in the PDB and superposed onto the anchor residues on the main model chain. In our case, this was not necessary because we chose to use a crystal structure with the whole backbone experimentally resolved (6P7G) as the template. After that, a set of independent models is created by the application. Loops are modeled first, evaluating their conformational energies, which are used to make a Boltzmann-weighted choice for the optimal loops to include in the model. Then, side chains are chosen from an extensive rotamer library generated by the LowModeMD approach, which was demonstrated to optimally search for energetic minima in complex multicomponent systems by exploiting short isothermal MD simulations and coupling them with all-atom energy minimizations. The intermediate models generated by these steps are then added with hydrogen atoms to complete the valence and are subjected to a further series of energy minimizations devoted to relieving eventual steric clashes. The final models are then ranked in a user-defined fashion, and in the present study, we chose to exploit the default electrostatic solvation energy, calculated using a Generalized Born/Volume Integral (GB/VI) method. For the molecular docking calculations, the program used was Glide [23] (from the Schrödinger suite, version 2021.3), using the Glide Standard Precision (Glide SP) method and exploiting the scoring function GlideScore.

## Data Availability

The data presented in this study are available from the corresponding author on request.

## Supplementary Materials

The following supporting information can be downloaded at: https://www.mdpi.com/article/10.3390/ijms241512285/s1.
