# Supplementary material for: Molecular Modeling Unveils the Effective Interaction of B-RAF Inhibitors with Rare B-RAF Insertion Variants
Source: Int J Mol Sci. 2023 Jul 31;24(15):12285. doi: 10.3390/ijms241512285 (PMC10418914; doi:10.3390/ijms241512285)
Supplement: Supplementary file 1 [file ijms-24-12285-s001.zip › ijms-2519498-supplementary.pdf]

Supplementary Material

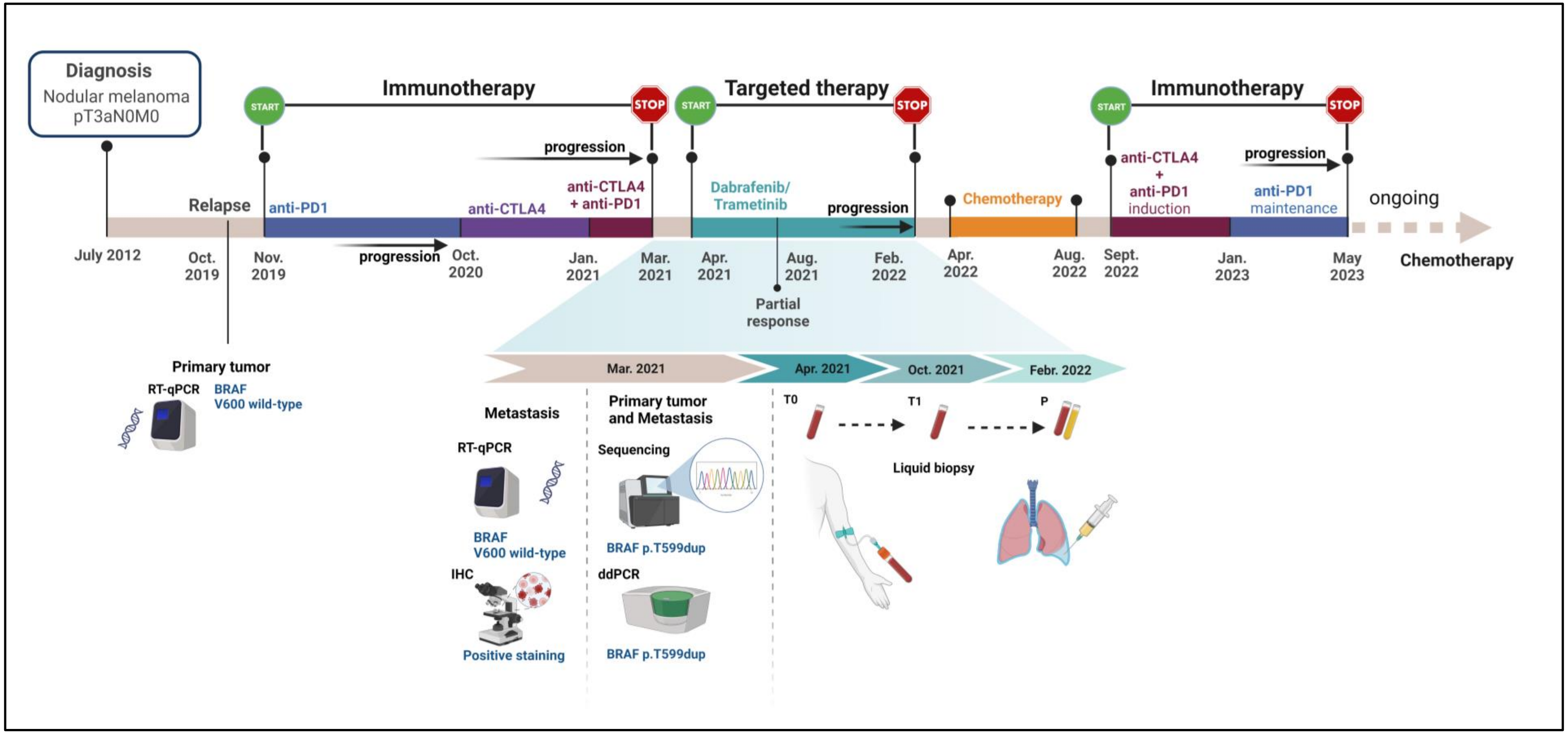

Supplementary Figure S1. Timeline of the disease management. Timeline describing the treatments administered to the patient, and the molecular analyses performed throughout the course of the disease. It was created with BioRender (https://biorender.com/).

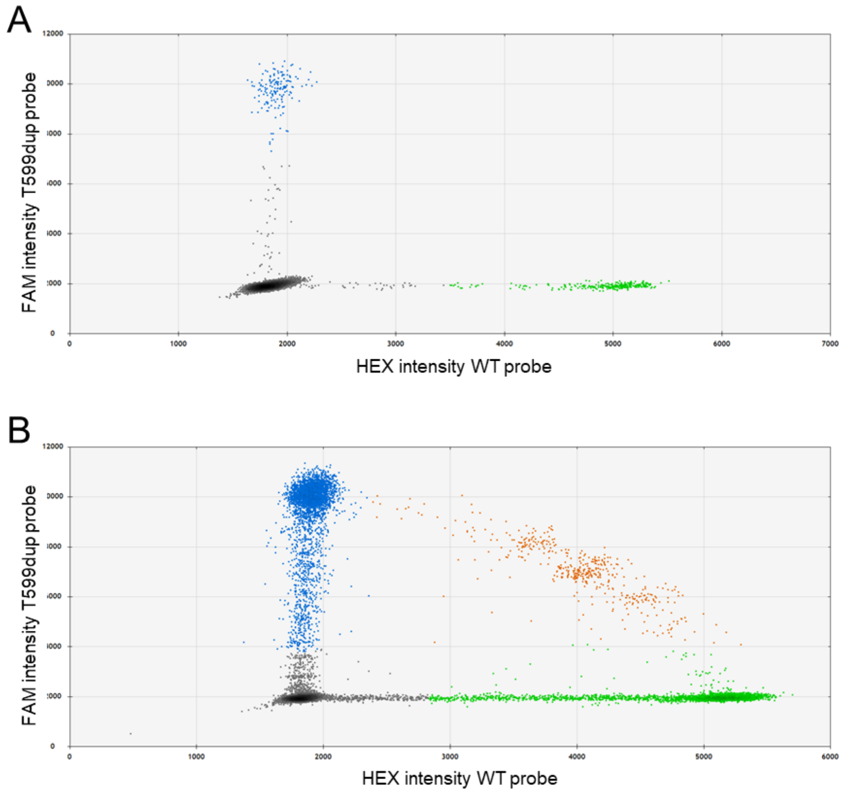

C

Sample	ddPCR MAF	NGS MAF
Primary tumor	34.0%	28.6%
Metastasis	48.0%	44.2%

Supplementary Figure S2. ddPCR and NGS analysis on primary and metastatic tumor tissue samples. 2D plot of ddPCR analysis (Custom PrimePCR ddPCR Mutation Assays for BRAF p.T599dup) of primary tumor (A) and metastatic (B) tissue samples. MAFs detected by ddPCR and NGS analyses are reported.

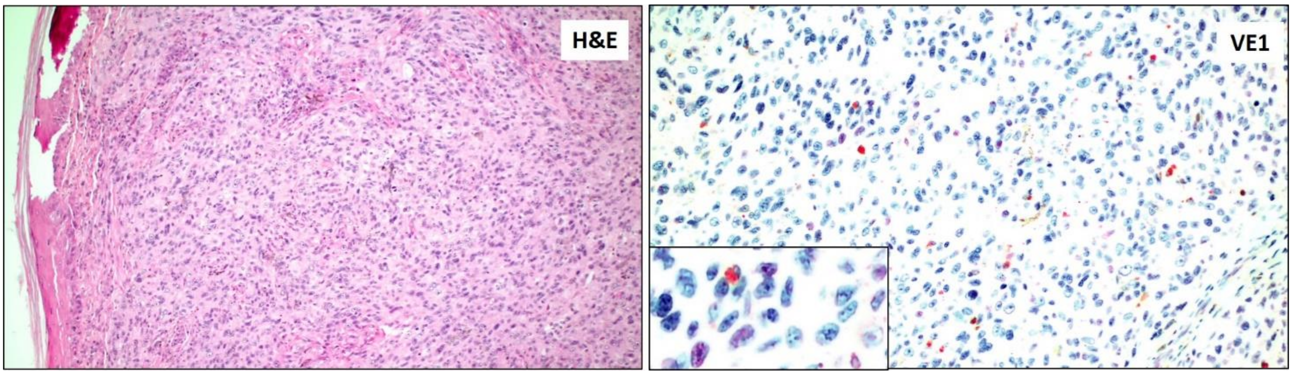

Supplementary Figure S3. B-RAF immunohistochemistry and sequencing of the pT3a primitive nodular melanoma. Staining with hematoxylin and eosin (H&E) and anti-B-RAF V600E monoclonal antibody (VE1) showing focal positivity for B-RAF mutant protein in melanoma cells.

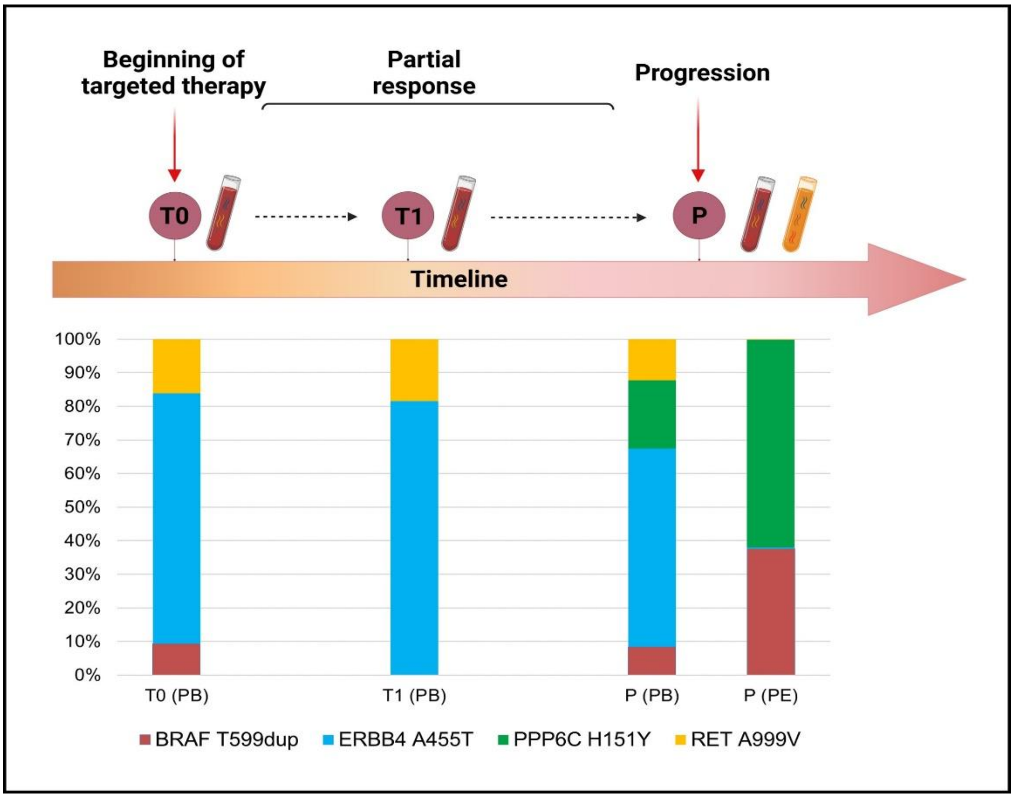

SNV	T0 (PB)	T1 (PB)	P (PB)	P (PE)
BRAF T599dup	0.64	UD	0.33	44.30
ERBB4 A455T	5.10	2.20	2.30	0.49
PPP6C H151Y	UD	UD	0.80	72.60
RET A999V	1.10	0.50	0.48	0.26

Supplementary Figure S4. Longitudinal tracking of the SNVs detected in the liquid biopsy. Contribute of the MAF (%) of each SNV over the total MAF (%) detected in each liquid biopsy sample. Time points of sample collection during monitoring: T0, before starting the therapy; T1, month 6 follow-up during clinically disease-free period; P, progression. Abbreviations: PB, peripheral blood; PE, pleural effusion; UD, undetectable. The timeline was created with BioRender (https://biorender.com/).

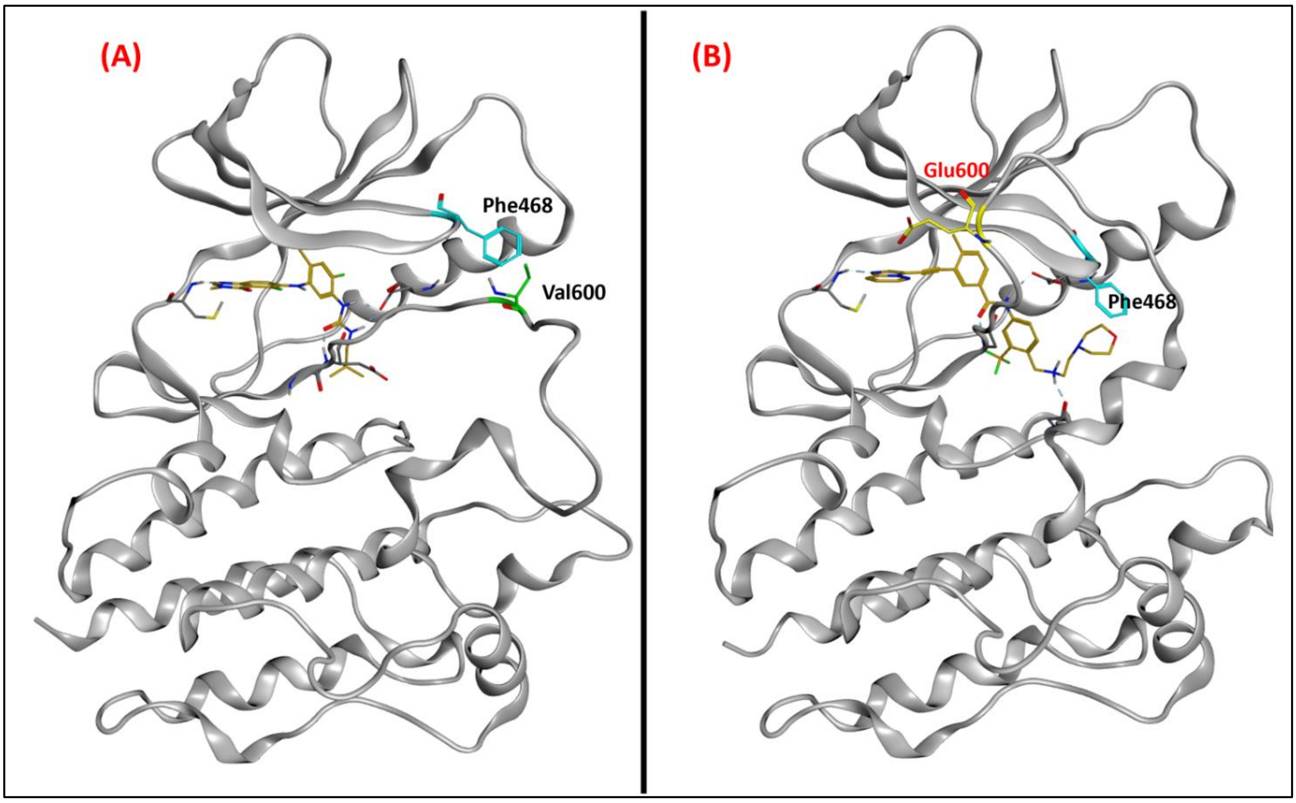

Supplementary Figure S5. Comparison between the WT (panel A, Protein Data Bank -PDB[18]- code: 7K0V) and the V600E variant (panel B, PDB code: 6PG7) of B-RAF protein kinase. As it can be seen, the contact between residue F468 (colored in cyan and labeled in both images) and V600 (colored in green in the left image) in the WT form of B-RAF is completely lost in the V600E variant (the E600 residue is colored in yellow and labeled in red in the right panel). This loss of contact induces a conformational change in B-RAF kinase, which becomes constitutively active, increasing the possibility of pathological outcomes.
